# Environmental Nontuberculous Mycobacteria in the Hawaiian Islands

**DOI:** 10.1371/journal.pntd.0005068

**Published:** 2016-10-25

**Authors:** Jennifer R. Honda, Nabeeh A. Hasan, Rebecca M. Davidson, Myra D. Williams, L. Elaine Epperson, Paul R. Reynolds, Terry Smith, Elena Iakhiaeva, Matthew J. Bankowski, Richard J. Wallace, Edward D. Chan, Joseph O. Falkinham, Michael Strong

**Affiliations:** 1 Division of Pulmonary Sciences and Critical Care Medicine, University of Colorado Denver, Anschutz Medical Campus, Aurora, Colorado, United States Of America; 2 Denver Veterans Affairs Medical Center, Denver, Colorado, United States Of America; 3 National Jewish Health, Denver, Colorado, United States Of America; 4 Virginia Tech, Blacksburg, Virginia, United States Of America; 5 Diagnostic Laboratory Services Inc., Aiea, Hawai’i, United States Of America; 6 Departments of Pathology and Tropical Medicine, Medical Microbiology and Pharmacology, John A. Burns School of Medicine, the University of Hawai’i at Manoa, Honolulu, Hawai’i, United States Of America; 7 University of Texas Health Science Center, Tyler, Texas, United States Of America; Institut Pasteur, FRANCE

## Abstract

Lung disease caused by nontuberculous mycobacteria (NTM) is an emerging infectious disease of global significance. Epidemiologic studies have shown the Hawaiian Islands have the highest prevalence of NTM lung infections in the United States. However, potential environmental reservoirs and species diversity have not been characterized. In this cross-sectional study, we describe molecular and phylogenetic comparisons of NTM isolated from 172 household plumbing biofilms and soil samples from 62 non-patient households and 15 respiratory specimens. Although non-uniform geographic sampling and availability of patient information were limitations, *Mycobacterium chimaera* was found to be the dominant species in both environmental and respiratory specimens. In contrast to previous studies from the continental U.S., no *Mycobacterium avium* was identified. *Mycobacterium intracellulare* was found only in respiratory specimens and a soil sample. We conclude that Hawai’i’s household water sources contain a unique composition of *Mycobacterium avium* complex (MAC), increasing our appreciation of NTM organisms of pulmonary importance in tropical environments.

## Author Summary

In the U.S., the Hawaiian Islands have the highest number of nontuberculous mycobacterial (NTM) lung disease cases per capita. The tropical climate, geographical isolation of the islands, and aquifer water sources may have influence such prevalence. Previous studies suggest that NTM thrive in water biofilms and soil. To broaden our understanding of potential environmental reservoirs and species composition of NTM in the Hawaiian Islands, we sampled environmental sites and examined patient isolates. Our recovery and identification of *Mycobacterium chimaera* and several other clinically relevant NTM species and the absence of *Mycobacterium avium* in both the indigenous environment and clinical specimens underscore the need for further studies to define the environmental factors that drive NTM lung disease and species composition in high prevalence locations such as the Hawaiian Islands.

## Introduction

Nontuberculous mycobacteria (NTM) are ubiquitous inhabitants of natural and human-engineered environments. To date, there are over 175 species of NTM with standing in nomenclature [1]. They range in virulence from benign environmental microorganisms to difficult-to-treat human pathogens [2]. Potentially pathogenic NTM have been documented in households, institutions (*i*.*e*., hospital premise plumbing), and soil [3]. In the continental United States (U.S.), household plumbing and environmental aerosols are thought to be important point sources of infection [4–8]. The most common NTM species to cause lung disease in the continental U.S. are those of the *Mycobacterium avium* complex (MAC)–slowly growing mycobacteria (SGM) that include *Mycobacterium avium* subsp. “*hominissuis*” and *Mycobacterium intracellulare* [9]. Clinically relevant environmental rapidly growing mycobacteria (RGM) include *Mycobacterium abscessus* subsp. *abscessus*, *massiliense*, and *bolletii* as well as the closely related species, *Mycobacterium chelonae* [10]. The current hypothesis is that NTM lung infections follow exposure to NTM from the home or other environmental source. [6]. Of interest, the predominant NTM species responsible for lung disease varies by geographic region, suggesting that environmental conditions (*e*.*g*., pH, oxygen, organic matter, and salinity) and the presence of other microorganisms influence NTM species numbers and diversity [11].

Despite the almost universal exposure to environmental NTM, pulmonary infections are relatively rare in otherwise healthy, non-bronchiectatic individuals and more common in individuals with abnormal lung architecture such as bronchiectasis and emphysema [12]. Nevertheless, it is important to identify the environmental niches that harbor potentially pathogenic NTM in geographical areas with a high prevalence of disease. In the U.S., the Hawaiian Islands were found to have the highest period prevalence of NTM lung disease (396 cases/100,000 persons for a total ten year time period) in a sampling of 2.3 million Medicare Part B beneficiaries enrolled from 1997 to 2007 [13]. In a follow-up study, spatial modeling revealed high-prevalence locations for NTM lung disease in this state [14]. The Hawaiian Islands also showed the highest age-adjusted mortality rates from NTM lung disease in the U.S., particularly in women over 55 years of age [15].

The high prevalence of NTM lung disease in the Hawaiian Islands provided the impetus to explore potential sources of infection and to determine the predominating NTM species in both environmental and clinical specimens. These islands are recognized for their unique island geology, flora, and fauna which are largely impacted by the tropical climate and isolation of the archipelago in the Pacific Ocean. Unlike most areas in the continental U.S. for which surface water serves as the primary public water source, underground aquifers provide water there. The Hawaiian Islands are also home to the highest number of elderly Asian-Pacific Islanders in the U.S.—a population previously recognized to be more susceptible to NTM infection [14]. To better understand NTM lung disease as a neglected tropical disease of emerging importance in this geographic area, the objective of the current work was to employ state-of-the-art molecular techniques to describe the indigenous NTM species composition in indoor and outdoor environments. A secondary objective was to analyze the genetic relatedness between the Hawaiian Island environmental NTM specimens (including 15 patient respiratory specimens) and continental U.S. NTM isolates.

## Methods

### Environmental sampling

In this cross-sectional study, we use the term “Hawaiian Islands” to designate the eight islands of the State of Hawai’i; the term “Hawai’i” refers to the youngest and largest island among the eight islands. Sample collection was conducted between December 2012 and January 2013. Samples were collected from 62 non-patient households located on the islands of Oahu, Molokai, Kauai, and Hawai’i. Detailed written instructions for collecting household water biofilms and soil samples were provided to local residents who volunteered to collect samples from their home as part of this study. As NTM are most commonly found in premise plumbing biofilms, samples were obtained by swabbing with sterile cotton-tipped applicators the inner surface of showerheads, kitchen and bath faucets, kitchen sink sprayers, refrigerator water dispensers, laundry room sinks, and shower drains [5, 6]. Samples from random sites in outdoor gardens or yards were also collected by clearing away surface leaves and other detritus and then scooping soil from the top five centimeters of ground into sterile 50 ml conical screw cap tubes as described [16].

### Pilot samples of patient isolates

Respiratory isolates of slowly-growing NTM recovered from 15 de-identified Oahu patients suspected of mycobacterial lung disease whose sputum had been submitted for mycobacterial culture were randomly selected from saved isolates at Diagnostic Laboratory Services, Inc. (Aiea, HI). *Mycobacterium tuberculosis* was not recovered in any of these sputum samples where NTM were isolated. As these were de-identified patient residual isolates, where only age and gender were noted from routinely ordered laboratory testing, Institutional Review Board (IRB) consent was waived. However, it was impossible to determine whether these patients met current American Thoracic Society/Infectious Disease Society of America (ATS/IDSA) diagnostic criteria for NTM pulmonary disease as private health information were delinked [9].

### Species and subspecies identification of NTM isolates by partial *rpoB* gene sequencing

Genome identification of environmental and patient NTM isolates was conducted through the amplification and sequencing of a 723 bp segment of the RNA polymerase beta subunit (*rpoB*) gene, also known as region 5 [17]. Sequences were trimmed for quality and compared against *rpoB* type strain sequences deposited in the National Center for Biotechnology Information (NCBI) GenBank using the BLAST algorithm. Definitions of species by single genes or spacer region were those of the Clinical Laboratory Standards Institute (CLSI) [18]. A sequence similarity cutoff of ≥ 98.3% was used to determine the species identification according to previously described cutoffs validated by studies of rapidly-growing mycobacteria [17]. The sequencing of NTM strains derived from patients was approved by the National Jewish Health Human Subject IRB.

### Non-Hawaiian Island NTM patient isolates

To determine whether NTM isolates from the Hawaiian Islands have shared sequence similarity with isolates obtained elsewhere, NTM type strains were included in genetic analyses. Type strains are denoted by superscript “T” and include *M*. *porcinum* CIP 105392 ^T^, *M*. *abscessus* subsp. *abscessus* ATCC 19977^T^, *M*. *abscessus* subsp. *bolletii* CIP 108541^T^, *M*. *chelonae* ATCC 35752^T^, and *M*. *chimaera* CIP 107892 ^T^. Additionally, 33 clinical respiratory isolates of *M*. *chimaera* (one per patient) from seven other states–Maryland, Texas, Louisiana, North Carolina, Oregon, Mississippi, and Arkansas–submitted for molecular identification to the *Nocardia/Mycobacteria* Research Laboratory, University of Texas Health Science Center, Tyler, Texas were included. Those isolates were identified to species by partial 16S rRNA and region 5 *rpoB* gene sequencing. This work was approved by the Human Subjects Committee of the University of Texas Health Science Center, Tyler, Texas.

### Nucleotide accession numbers

Partial *rpoB* gene sequences from 166 Hawaiian Island NTM isolates and 33 *M*. *chimaera* isolates from the continental U.S. were deposited in the GenBank nucleotide database. The GenBank accession numbers for type strain and representative isolate *rpoB* gene sequences of *M*. *porcinum*, *M*. *abscessus*, *M*. *chelonae*, and *M*. *chimaera* from NCBI are also listed in S1 Table

### Phylogenetic and sequence variant network analyses

Partial *rpoB* sequences of respiratory and environmental NTM isolates (n = 166) were aligned using MUSCLE [19] and sequence alignments were trimmed to remove missing data from the ends of the final alignment. Phylogenetic trees were generated using the neighbor-joining method based on the number of nucleotide differences and uniform rates among sites while omitting any sites in the alignment with gaps or missing data in MEGA version 6 [20].

For *rpoB* sequence variant analyses, only sequences greater than 600bp and with no ambiguous base calls were included. Sequences were grouped by species and compared to selected type and non-type strain sequences from NCBI. The PopART population genetics software was used to examine intraspecies sequence variation, generate species-specific *rpoB* sequence variant networks, and label isolates by isolation source: *i*.*e*., kitchen, bathroom, soil, patient [21]. For the *M*. *porcinum*, *M*. *abscessus*, and *M*. *chelonae* analyses, the environmental Hawaiian Island isolates and both type and non-type strains were included. For the *M*. *chimaera* analysis, environmental and clinical Hawaiian Island isolates, type, and non-type strains, as well as clinical isolates from seven states across the continental U.S. were included.

### Statistical analyses

Statistical analyses were performed using R version 2.13.2 [22]. Fisher’s Exact Tests were used to evaluate differences in proportions of NTM species or species groups between household areas (*i*.*e*., bathroom, kitchen, and soil) or sample type (biofilm and soil).

## Results

### Environmental Areas Sampled

**Fig 1:**
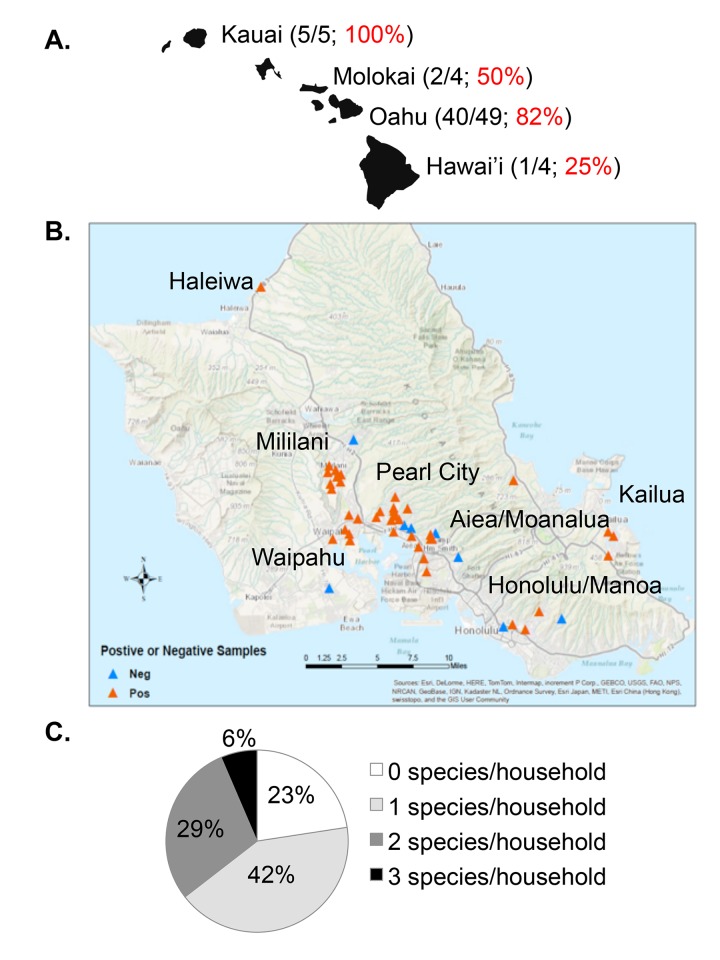
Environmental sampling for NTM. A) Biofilm swabs and soil samples were collected from 62 households on four of eight principal Hawaiian Islands. The numbers and proportions of total households from which NTM were recovered are shown. B) Locations of households sampled in towns across the island of Oahu. Colored triangles indicate sampling sites with biofilm or soil samples that were positive for presence of NTM (red) or negative for NTM (blue). C) The number of NTM species recovered from each household was calculated. Shown are the proportions of households harboring zero NTM species/household, one NTM species/household, two NTM species/household or three different NTM species/household.

From a total of 62 households across four islands **(Fig 1A)**, a total of 172 biofilm and soil samples were collected. The majority of the samples (n = 134, 78%) were collected from Oahu and included 35 showerheads (26%), 41 kitchen faucets (31%), 6 bathroom sink faucets (4%), 2 refrigerator water taps (1%), 3 other biofilm samples from laundry room faucets (2%), and 47 soil samples (35%). The remaining 38 samples (22%) were collected from 13 households on the neighbor islands.

Among all 172 biofilm and soil samples collected from the 62 households, NTM were isolated from 44% of samples (75/172) (**Table 1).** NTM were identified in nearly half of the samples on Oahu (65/134, 49%) and in approximately a quarter of samples from the neighbor islands (10/38, 26%). Overall, the NTM culture positivity rate for biofilms was 59% (67/113), which was significantly greater than for soil (14%, 8/59; p = 6.0x10^-9^).

**Table 1:** Description of household biofilm and yard/garden soil sampling and proportions of samples that were NTM culture-positive.

	Oahu			Kauai			Molokai			Hawai’i		
	n = 49			n = 5			n = 4			n = 4		
	#	#	%	#	#	%	#	#	%	#	#	%
	samples	NTM+	NTM+	samples	NTM+	NTM+	samples	NTM+	NTM+	samples	NTM+	NTM+
Showerhead	35	24	69%	5	4	80%	5	1	20%	2	0	0%
Kitchen	41	24	59%	3	2	67%	5	1	20%	3	1	33%
Bathroom	6	4	67%	ND	ND	ND	ND	ND	ND	2	0	0%
Refrigerator tap	2	2	100%	ND	ND	ND	ND	ND	ND	ND	ND	ND
Other	3	3	100%	1	1	100%	ND	ND	ND	ND	ND	ND
**Total Biofilms**	**87**	**57**	**66%**	**9**	**7**	**78%**	**10**	**2**	**20%**	**7**	**1**	**14%**
Soil	47	8	17%	3	0	0%	5	0	0%	4	0	0%
**Total**	**134**	**65**	**49%**	**12**	**7**	**58%**	**15**	**2**	**13%**	**11**	**1**	**9%**

Total households sampled = 62

Total number of samples collected from households = 172 (n = 113 biofilm samples and n = 59 soil samples)

Total NTM culture positive samples = 75/172 (44%)

Other includes laundry and bedroom faucets

ND = Not done

### NTM Recovered by Household

The majority of the environmental samples collected were from 49 households in seven different towns on Oahu, the most populated island (**Fig 1B**). NTM were recovered by culture from 82% of the Oahu households (**Fig 1A**). For the neighboring islands, NTM were also recovered in households on Kauai, Molokai, and Hawai’i (**Fig 1A**). Among the 62 collective households sampled in this study, only 14 had no NTM isolated (23%). However, the number of households with one, two, and three different NTM species isolated were 26/62 (42%), 18/62 (29%), and 4/62 (6%), respectively **(Fig 1C)**. Overall, the majority of households (43/62, 69%) had at least one clinically relevant species of MAC, *M*. *abscessus* subsp., or *M*. *chelonae*—**(Table 2)**.

**Table 2:** NTM species and combinations of species recovered from 62 households sampled from the Hawaiian Islands (includes biofilm and soil samples).

NTM species	# of households	# of NTM species per household	High clinical prevalence NTM species*
None	14	0	N/A
*M*. *avium* complex (MAC)**	16	1	yes
*M*. *abscessus* or *M*. *chelonae*	5	1	yes
*M*. *porcinum*	3	1	no
Other NTM	2	1	no
*M*. *abscessus* or *M*. *chelonae* + other NTM	4	2	yes
MAC + *M*. *abscessus* or *M*. *chelonae*	6	2	yes
MAC + *M*. *porcinum*	4	2	yes
MAC + other NTM	4	2	yes
MAC + *M*. *abscessus* + other NTM	4	3	yes

* NTM species associated with high clinical prevalence in previous epidemiological studies. Ref 10, 11.

** *M*. *avium* complex (MAC) includes *M*. *chimaera* and *M*. *intracellulare*. *M*. *intracellulare* was not recovered from biofilm samples, only a soil sample. *M*. *avium* was not recovered from any of the species examined.

### NTM Recovery in Non-Household Samples

To determine the diversity of NTM in non-household sites, 13 environmental samples (n = 7 biofilm and n = 6 soil) were collected from eight public areas on Oahu and Kauai **(Table 3).** On Oahu, a total of six biofilms from public sites were collected including gymnasium showerheads and water fountain taps. Four soil samples were also collected from public sites on Oahu. Two water biofilm and two soil samples were collected from public sites on Kauai. One Oahu public site soil sample contained *M*. *chimaera* (1/6 = 17%) and one biofilm sample contained *M*. *chelonae* (1/7 = 14%), but the majority (5/13 = 38%) yielded other RGM species (*i*.*e*., *M*. *barrassiae*, *M*. *alvei*, and *M*. *septicum*). *RpoB* sequences from four distinct isolates did not have NCBI database matches above 95% sequence identity, suggesting they represent novel species.

**Table 3:** Description and NTM recovery in non-household samples.

	Type of sample:	Source of sample:	NTM species identified:	Town, Island:
1.	a) Soil	a) Gym	a) None	Pearl City, Oahu
	b) Biofilm swab	b) Hot tub (n = 1)	b) None	
2.	a) Soil	a) Fruit cannery	a) *M*. *chimaera*	Wahiawa, Oahu
	b) Biofilm swab	b) Water fountain (n = 1)	b) *M*. *gordonae*	
			c) Potential novel species *	
3.	a) Soil	a) Cemetery	a) *M*. *barrassiae*	Waipio, Oahu
	b) Biofilm swab	b) Outdoor faucet (n = 1)	b) None	
4.	a) Soil	a) Gym^^^	a) Potential novel species*	Hawai’i Kai, Oahu
	b) Biofilm swab	b) Water fountain (n = 1)	b) Potential novel species**	
	c) Biofilm swab	c) Showerhead (n = 1)	c) None	
			d) None	
5.	Biofilm swab	Water fountain at a pier (n = 1)	*M*. *gordonae*	Honolulu, Oahu
6.	Biofilm swab	Water fountain at tourist stop (n = 1)	*M*. *chelonae*	Waimea, Kauai
7.	Soil	Tourist stop (n = 1)	a) *M*. *alvei*	Waimea, Kauai
			b) Potential novel species***	
8.	Soil	Grotto (n = 1)	*M*. *septicum*	Kapa’a, Kauai

* 93% identity to *M*. *gadium*

** 90% identity to *M*. *jacuzzii*

*** 94% identity to *M*. *alvei* and *M*. *fortuitum*.

^^^ The only non-household site out of eight from which more than one household biofilm sample was collected.

### Spectrum of NTM Species Identified from Environmental Samples

**Fig 2:**
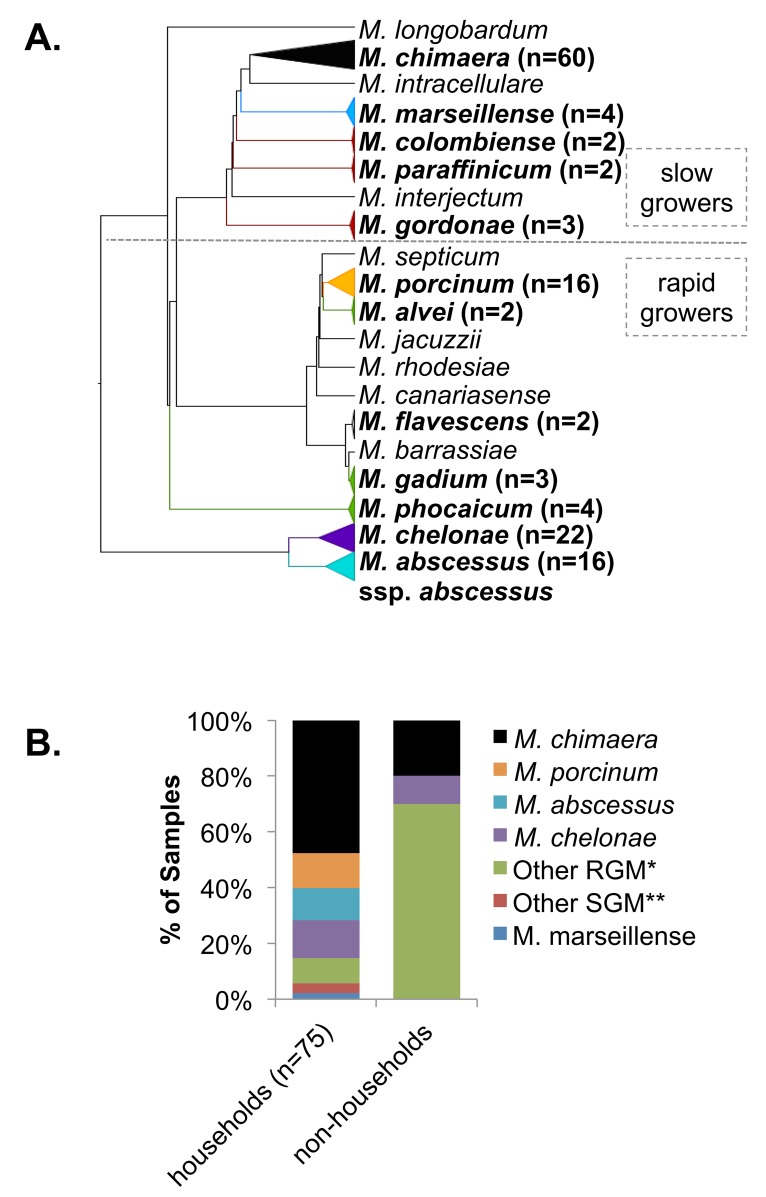
Diversity and frequency of NTM species recovered from environmental samples. A) Phylogenetic analysis was performed from a multiple sequence alignment of partial *rpoB* sequences to illustrate the distribution of SGM and RGM isolates identified among environmental samples. Bolded names indicate NTM species in which more than one isolate was identified across the sample set. B) Proportions of samples positive for NTM species are shown for households (n = 75) and non-household sites (n = 9).

Among the 75 environmental samples from the households that were NTM culture-positive, 20 different NTM species were identified (**Fig 2A**) and 17% (13/75) grew out multiple NTM species. The most common species recovered from households were MAC organisms with *M*. *chimaera* being the predominant species (42/75, 56%) **(Fig 2B, left).** The next most frequently isolated species were *M*. *chelonae* (12/75, 12%) and *M*. *porcinum* (11/75, 11%). All isolates of *M*. *abscessus* were confirmed as *M*. *abscessus* subsp. *abscessus* (10/75, 10%) [23, 24]. Less frequently isolated NTM species (<10%) included *M*. *phocaicum*, *M*. *gadium*, *M*. *alvei*, *M*. *gordonae*, *M*. *paraffinicum*, *M*. *marseillense*, and *M*. *colombiense*. No isolates of *M*. *avium* or *M*. *intracellulare* were recovered from household biofilm samples, though *M*. *intracellulare* was isolated from a single soil sample. While *M*. *chimaera* and *M*. *chelonae* were identified in non-household samples, the majority classified as other NTM included potentially novel species **(Fig 2B, right).**

### NTM Predominating in Household Locations

**Fig 3:**
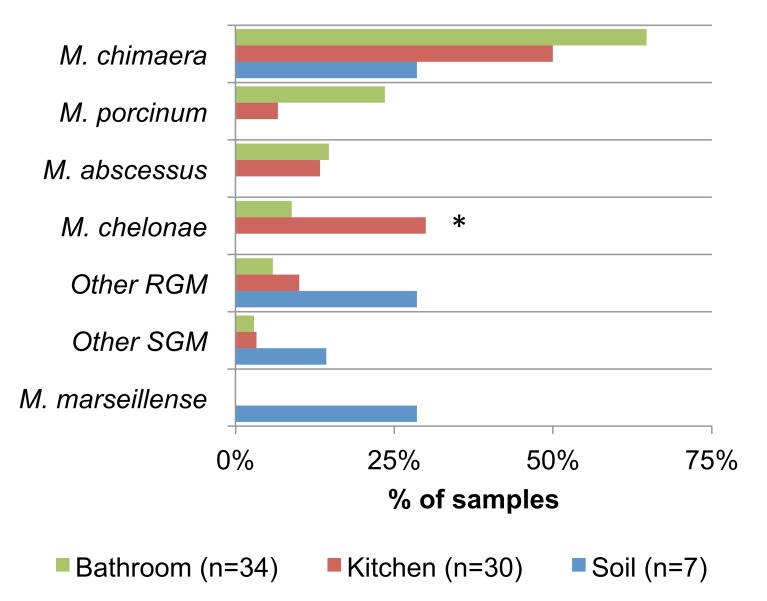
Household distribution of NTM species. Proportions of household samples harboring various NTM species are shown. Statistical comparisons between household locations were performed by Fisher’s exact tests (*p = 0.05).

To determine whether NTM were present in particular household locations, the frequencies of NTM recovery between bathroom biofilms, kitchen biofilms, and soil were compared **(Fig 3).** *M*. *chimaera* was frequently identified from both bathroom (22/34, 65%) and kitchen (15/30, 50%) biofilms and was also identified in soil (2/7, 29%). *M*. *porcinum* was overrepresented in bathroom (8/34, 24%) compared to kitchen biofilms (2/30, 7%; p = 0.09), while *M*. *chelonae* was significantly more common in kitchen (9/30, 35%) compared to bathroom biofilms (3/34, 9%; *p = 0.05). *M*. *abscessus* was observed in similar proportions between bathroom (5/34, 15%) and kitchen (4/30, 13%) biofilms. *M*. *porcinum*, *M*. *chelonae*, and *M*. *abscessus* were not recovered from soil. *M*. *marseillense* was recovered only from soil and not identified in any of the household biofilm samples. NTM species that showed low prevalence in our study (*i*.*e*., one isolate per species identified in the entire sample set and labeled “other RGM” and “other SGM”) were primarily isolated from soil samples.

### Genetic Diversity of Environmental RGM Isolates

**Fig 4:**
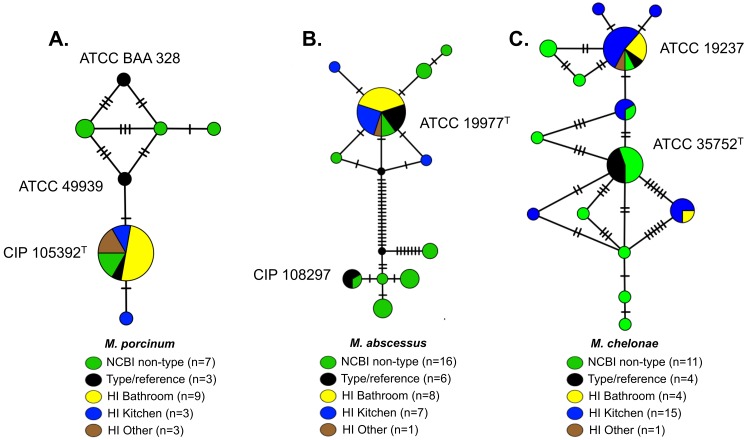
Distributions of *rpoB* sequence variants detected among Hawaiian Island environmental isolates of *M*. *porcinum*, *M*. *abscessus*, and *M*. *chelonae* compared to type strains. Sequence variant networks were created based on alignments of partial *rpoB* gene sequences for: A) *M*. *porcinum* (n = 25 total sequences, out of 615 positions) B) *M*. *abscessus* (n = 38 total sequences, out of 610 positions) and C) *M*. *chelonae* (n = 35 total sequences, out of 613 positions). Pie charts were used to indicate the distribution of isolates from different sources sharing an identical *rpoB* variant. Colors reflect distinct isolate sources. Hash marks indicate SNP differences between adjacent isolate subgroups. Isolates per *rpoB* variant (n = X) are specified for each variant occurring in more than one isolate. Type strains are indicated next to their designated variant group and are denoted by superscript “T.”

To examine population diversity among RGM isolates from individual households, *rpoB* sequences of *M*. *porcinum*, *M*. *abscessus*, and *M*. *chelonae* were analyzed **(Fig 4).** Type and non-type strain *rpoB* sequences were included for comparison. In the *M*. *porcinum* dataset (n = 25 sequences), a total of seven sequence variants were identified **(Fig 4A)**. All isolates from the bathroom, kitchen, and outside faucets were in the same sequence variant group as the *M*. *porcinum* type strain, CIP 105392^T^, except for one kitchen isolate that contained a single SNP difference. Among all *M*. *abscessus* sequences (Hawaiian Island and type/reference strains; n = 38), six sequence variants of subsp. *abscessus*, four variants of subsp. *massiliense*, and one of subsp.*bolletii* **(Fig 4B)** were identified. Environmental *M*. *abscessus* isolates grouped with other *M*. *abscessus* subsp. *abscessus* and the majority of *M*. *abscessus* isolates (13/16 = 81%) shared an identical *rpoB* sequence with the type strain, ATCC 19977^T^. Three additional isolates differed by one SNP each from the ATCC 19977^T^ type strain. Finally, *M*. *chelonae* isolates **(Fig 4C)** showed the greatest *rpoB* sequence variation with a total of 14 *rpoB* sequence variants. Hawaiian Island *M*. *chelonae* isolates fell into seven *rpoB* sequence variant groups, but the majority (15/20 = 80%) fell into two main subgroups: one group (6/15 and 40%) sharing the *M*. *chelonae* ATCC 19237 *rpoB* variant and a second group (5/15 and 33%) related to the *M*. *chelonae* ATCC 35752^T^ *rpoB* variant.

### Genetic Diversity of Environmental and Clinical *M*. *chimaera* Isolates

As the majority of the Hawaiian Island environmental NTM isolates from this study were *M*. *chimaera*, 15 random respiratory SGM isolates from Oahu patients presenting to a pulmonary clinic with suspected mycobacterial lung disease were used as pilot samples to evaluate for the presence of *M*. *chimaera* in clinical specimens. As a group, the median age of the 15 patients was 75 years (95% CI, 68; 81 years) and 67% (10/15) were female **(Table 4).** Ten isolates were identified as *M*. *chimaera* (10/15, 67%) four as *M*. *intracellulare* (4/15, 27%), and one as *M*. *marseillense* (1/15, 6%). Of the ten patients with *M*. *chimaera*, 60% (6/10) were female. All four patients with *M*. *intracellulare* were female (100%; 5/5) and the patient with *M*. *marseillense* was male **(Table 4).** *M*. *avium* was not identified from any of the Oahu clinical isolates.

**Table 4:** NTM species identified and demographic information of 15 pilot Oahu clinical isolates.

	NTM Identified:	Age (yrs)	Gender
1.	*M*. *chimaera*	57	F
2.	*M*. *chimaera*	54	F
3.	*M*. *chimaera*	73	F
4.	*M*. *chimaera*	67	F
5.	*M*. *chimaera*	89	F
6.	*M*. *chimaera*	65	F
7.	*M*. *chimaera*	74	M
8.	*M*. *chimaera*	80	M
9.	*M*. *chimaera*	87	M
10.	*M*. *chimaera*	79	M
11	*M*. *intracellulare*	90	F
12.	*M*. *intracellulare*	67	F
13.	*M*. *intracellulare*	70	F
14.	*M*. *intracellulare*	87	F
15.	*M*. *marseillense*	78	M

**Fig 5:**
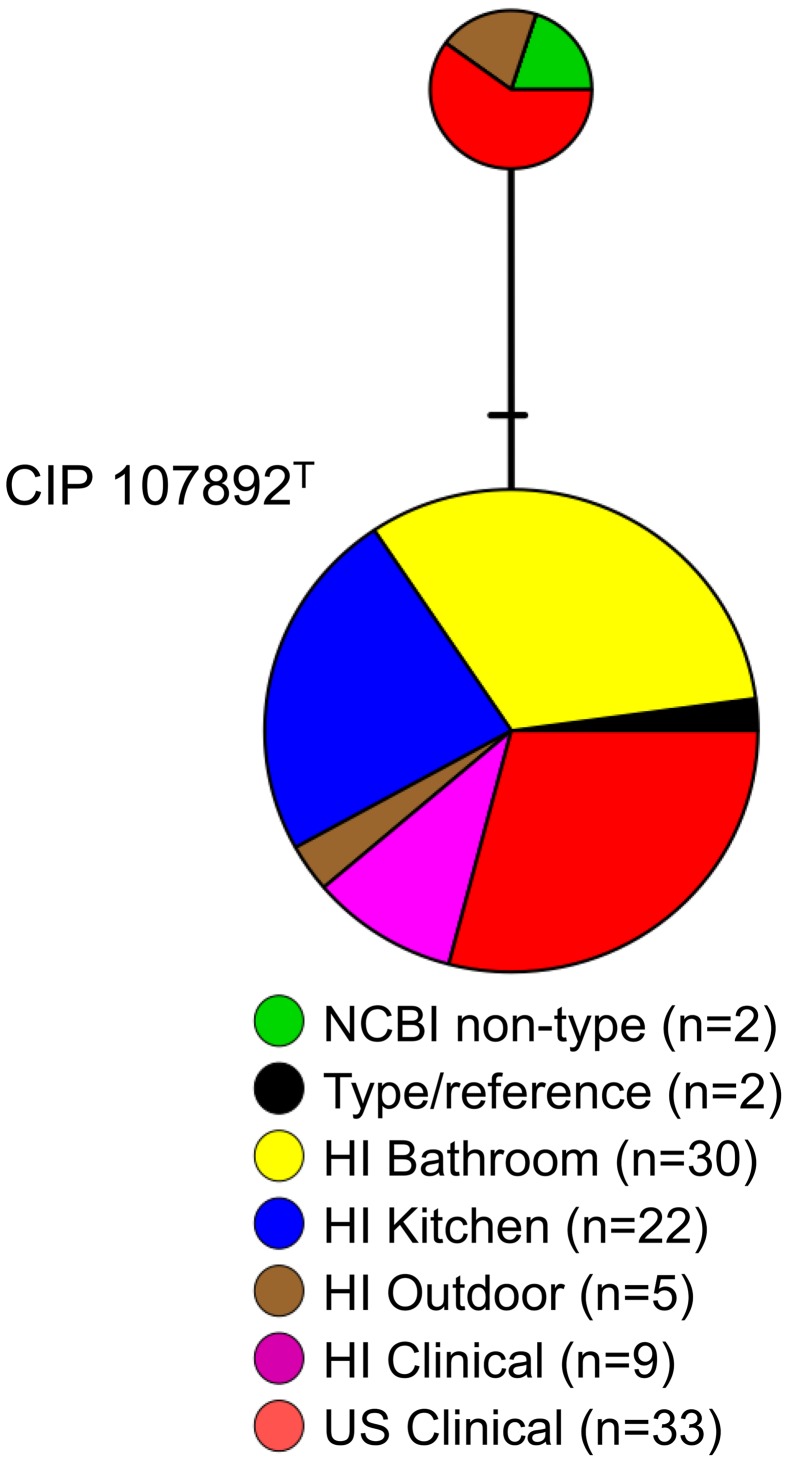
Distribution of *rpoB* sequence variants among Hawaiian Island environmental and clinical isolates of *M*. *chimaera* compared to type strains. A sequence variant network was created based on an alignment of partial *rpoB* gene sequences (n = 103 total sequences, out of 591 positions) including environmental and Oahu clinical isolates. Hash marks indicate SNP differences between adjacent isolate subgroups (circles). Isolates per *rpoB* variant (n = X) are specified for each observed variant. Type and non-type strain sequences and random clinical isolates from the continental U.S. were included for comparison. The *M*. *chimaera* type strain is denoted by superscript “T”.

To measure the genetic similarity among a diverse collection of environmental and clinical *M*. *chimaera*, we analyzed *rpoB* sequence variation between the 57 Hawaiian Island environmental *M*. *chimaera* isolates and the 10 Oahu respiratory *M*. *chimaera* isolates. However, the *rpoB* sequence of one clinical *M*. *chimaera* isolate was excluded from these analyses due to the presence of ambiguous bases. Also included were NCBI non-type strains (n = 2), type strains (n = 2), and other *M*. *chimaera* respiratory isolates (n = 33) from seven states in the continental U.S. In total, 103 *M*. *chimaera* sequences were analyzed and only two *rpoB* sequence variants were observed **(Fig 5)**. The larger variant subgroup comprised over 90% of the isolates including all of the Oahu respiratory and biofilm *M*. *chimaera* isolates. This group also contained the majority of continental U.S. clinical isolates and the CIP107892^T^ type strain. The smaller variant subgroup contained continental U.S. clinical isolates, non-type strains from NCBI, and Hawaiian Island soil isolates.

## Discussion

To our knowledge, this is the first assessment of environmental NTM prevalence and species composition in the Hawaiian Islands. This archipelago is approximately halfway between the continental U.S. and Asia; thus, one might speculate that the spectrum of NTM observed mirrors the results from other environmental studies from the continental U.S. or Asia. Due to the prevalence of *M*. *avium* subsp. *“hominissuis”* reported in studies from the continental U.S. and Japan [25–27], we suspected this species would be prevalent in Hawaiian Island household biofilms and patient samples; however, it was seemingly absent, at least in the samples examined in this study. In general, NTM are rare in groundwater [29] whereas *M*. *avium* subsp. “*hominissuis”* has been isolated from surface water sources [28]. Aquifers provide most of the drinking water in the Hawaiian Islands [30] which may be one reason for the lack of *M*. *avium* detection in our samples. However, given the widespread prevalence of *M*. *chimaera* and the RGM in Hawaiian Island household biofilms, local aquifers may be a potential reservoir for *M*. *chimaera* and other NTM. Future studies are needed to examine this hypothesis.

To date, species diversity assessments of environmental NTM in other tropical Pacific Islands remains scant. A recent study described the identification of the *M*. *fortuitum* complex in Polynesian residents with suspected tuberculosis [31] and other reports from the area highlight NTM-associated skin disease [32, 33]. On Australia, *M*. *intracellulare* was reported as the species responsible for most lung disease cases and yet only *M*. *avium* subsp. “*hominissuis”*, *M*. *kansasii*, and *M*. *abscessus* isolates had a species that match between patients and their household water system [34, 35].

An unexpected finding of this study was the frequent identification of *M*. *chimaera* from both the environmental samples collected from bathroom, kitchen, and soil samples (Fig 3) and patient isolates with suspected mycobacterial lung disease. Although the number of patient isolates was small and their disease status were not known, the correspondence between the high proportion of both environmental and clinical *M*. *chimaera* isolates is intriguing and offers direction for future investigations. *M*. *chimaera* was first described in 2004 [36] and was recently reported to cause health-care associated infections after open-heart surgery with the use of heater-cooler units [37, 38]. As this is a relatively newly described species, there are no simple methods to differentiate *M*. *chimaera* from *M*. *intracellulare*. Furthermore, low frequency of presence in lung samples of patients from Germany, Italy, Zambia, and China [39–41] is most likely due to its misidentification as *M*. *intracellulare*. A greater adoption of more refined molecular methods to distinguish *M*. *chimaera* from *M*. *intracellulare* has facilitated the more precise speciation of *M*. *chimaera* (33). In a previous U.S. study, water biofilm isolates originally reported as *M*. *intracellulare*, proved to be *M*. *chimaera* or other MAC-X [4]. Provisionally, it appears that the main environmental source of *M*. *chimaera* in the Hawaiian Islands are water biofilms and less from the soil (Fig 3), whereas *M*. *intracellulare* was absent in water biofilms and only recovered from soil, consistent with the finding of others 4 (Fig 3, other SGM). Soil should also be regarded as a potential reservoir for *M*. *marseillense*.

Among our environmental samples, *M*. *porcinum*, *M*. *chelonae*, and *M*. *abscessus* were the most frequently identified RGM species. The *M*. *fortuitum* complex including *M*. *porcinum* were found to comprise the majority of clinical isolates examined in French Polynesia (42/87, 48%) using partial *rpoB* gene sequencing [31]. Of these, *M*. *porcinum* was identified in three patients who fulfilled ATS criteria for NTM lung disease. To our knowledge, *M*. *porcinum* infections have not yet been reported in the Hawaiian Islands, but the organism has been isolated from water supplies in other U.S. areas (*e*.*g*., Texas) [42, 43]. *M*. *abscessus* was recently associated with an outbreak in cystic fibrosis patients at a hospital in Hawai’i [44]. *M*. *chelonae* infection was reported in a case study of an individual from Hawai’i after laser *in situ* keratomileusis (LASIK) surgery [45]. It is important to mention that among the environmental samples in this study, these particular RGM were more commonly identified in bathroom and kitchen biofilm samples and absent from soil (Fig 3), suggesting a preferential environmental niche for these particular RGM species.

Phylogenetic analyses were performed to evaluate whether the genetic diversity among environmental NTM species identified from the Hawaiian Island samples differed from those collected from the continental U.S. A relatively high genetic diversity among *M*. *chelonae* was observed with four major *rpoB* subgroups present, while most isolates of *M*. *porcinum* and *M*. *abscessus* belonged to one major genetic group per species (Fig 4). The presence of only two genetic subtypes of *M*. *chimaera* among a geographically diverse population of environmental and suspect respiratory Oahu specimens, as well as clinical isolates from seven other states in the continental U.S. suggests a low level of genetic divergence occurring in this species (Fig 5). Whole genome sequence comparisons will be necessary to improve our understanding of the genetic relationships between environmental and respiratory populations of *M*. *chimaera*.

This study has some limitations including the following in methodology: *(i)* we were unable to consistently collect a large number of samples from the same indoor sites for each participating household, *(ii)* a sampling bias exists as the majority of samples were collected from Oahu (home to the majority of the state’s population) with only a few household samples collected from the less populated Molokai, Kauai, and Hawai’i and none from Kaho’olawe, Maui, Lanai, or Ni’ihau, and *(iii)* instead of a single person conducting all environmental sampling, household areas were sampled by local citizens, which added a layer of non-equivalency to the process of sample collection. To reduce non-uniformity in the collection process, we applied a well-accepted citizen science approach to minimize variability introduced by handling of samples by different people [46]. Although we cannot be certain our findings represent the true geographic diversity of NTM in the Hawaiian Islands, this work describes the largest study of environmental NTM in this geographic area with a documented high NTM disease burden. We would advocate for a larger, randomized systematic study of the distribution of environmental NTM in future work. To the best of our knowledge, all environmental samples were from households whose occupants are not known to have NTM lung disease; thus, it will be imperative to sample NTM patient households in a larger future study especially as a more thorough comparison of prevalence and numbers of NTM species in patients and their local environment can be assessed. We were also unable to confirm that the clinical isolates used in this study were etiological agents of respiratory disease or due to benign colonization from environmental exposures. Additionally, this pilot clinical isolate panel did not contain any RGM. Nevertheless, the observation that *M*. *chimaera* was the most common species in both environmental and clinical isolates examined suggests the possibility of environmental exposures and clinical NTM lung disease. To determine whether NTM in the household environment contributes to clinical disease, we hope to initiate a large-scale genomic study of matched household and clinical NTM isolates from NTM-infected Hawai’i patients who fulfill ATS/IDSA criteria for lung disease. Undoubtedly, the data collectively presented in this study will be valuable in guiding the design of a more comprehensive study.

In summary, this study describes environmental sampling, microbiological selection, and molecular identification to determine the NTM species diversity in the Hawaiian Island environment. The observation that *M*. *chimaera* was the most common NTM species identified in both our Hawai’i environmental samples as well as in a small sampling of respiratory specimens from patients with suspected mycobacterial lung disease suggests that *M*. *chimaera* may be an important environmentally acquired respiratory pathogen. Furthermore, *M*. *chimaera* may be unique in prevalence in tropical climates such as Hawai’i. Additional studies with systematic collection of matched environmental and respiratory specimens, high-resolution genotyping methods, and correlation with demographic and epidemiological data (*i*.*e*. age, gender together with ethnicity and host risk and genetic factors) will be necessary to further characterize this observation and the important clinical implications.

## Supporting Information

S1 Checklist STROBE checklist. (DOC)

S1 Table Accession Numbers For Study Isolates. *Mycobacteria* isolates derived from Hawaiian Island and Continental U.S.A. clinical and household specimens. Species identification and NCBI GenBank accession numbers are provided for each isolate.
(PDF)
