# Correction: Environmental Nontuberculous Mycobacteria in the Hawaiian Islands

**DOI:** 10.1371/journal.pntd.0005200

**Published:** 2016-12-02

**Authors:** Jennifer R. Honda, Nabeeh A. Hasan, Rebecca M. Davidson, Myra D. Williams, L. Elaine Epperson, Paul R. Reynolds, Terry Smith, Elena Iakhiaeva, Matthew J. Bankowski, Richard J. Wallace, Edward D. Chan, Joseph O. Falkinham, Michael Strong

There are errors in the author affiliations. The affiliations should appear as shown here:

Terry Smith^7^, Elena Iakhiaeva^7^

**7** University of Texas Health Science Center, Tyler, Texas, United States of America
