# Supplementary material for: Environmental Nontuberculous Mycobacteria in the Hawaiian Islands
Source: PLoS Negl Trop Dis. 2016 Oct 25;10(10):e0005068. doi: 10.1371/journal.pntd.0005068 (PMC5079566; doi:10.1371/journal.pntd.0005068)
Supplement: S1 Checklist — (DOC) [file pntd.0005068.s001.doc]

STROBE Statement—Checklist of items that should be included in reports of ***cross-sectional studies***

|  | Item No | Recommendation |
| --- | --- | --- |
| **Title and abstract** | 1 | (*a*) Indicate the study’s design with a commonly used term in the title or the abstract |
|  |  | (*b*) Provide in the abstract an informative and balanced summary of what was done and what was found  Response: The word “cross-sectional” as been added to the abstract (line 67, tracked version and line 361, tracked version) |
| Introduction | | |
| Background/rationale | 2 | Explain the scientific background and rationale for the investigation being reported |
|  |  | Response: Included in revision (line 278-282 tracked version) |
| Objectives | 3 | State specific objectives, including any prespecified hypotheses  Response: Included in revision (line 286-355 tracked version) |
| Methods | | |
| Study design | 4 | Present key elements of study design early in the paper  Response: Included in revision; abstract (line 67, tracked version and line 361, tracked version) |
| Setting | 5 | Describe the setting, locations, and relevant dates, including periods of recruitment, exposure, follow-up, and data collection  Response: Included across the Methods section of revision. |
| Participants | 6 | (*a*) Give the eligibility criteria, and the sources and methods of selection of participants  Response: 15 clinical isolates of NTM were used in this study solely as pilot samples to begin to study *M. chimaera* in Hawai’i (lines 367, 1064-1073 tracked version). |
| Variables | 7 | Clearly define all outcomes, exposures, predictors, potential confounders, and effect modifiers. Give diagnostic criteria, if applicable  Response: N/A |
| Data sources/ measurement | 8* | For each variable of interest, give sources of data and details of methods of assessment (measurement). Describe comparability of assessment methods if there is more than one group  Response: Data assessments used in this study are detailed on lines 649-663 (tracked version). |
| Bias | 9 | Describe any efforts to address potential sources of bias  Response: Limitations are addressed in the revision; lines 1328-1381 (tracked version). |
| Study size | 10 | Explain how the study size was arrived at  Response: This study was limited due to funding constraints. Thus, only 15 clinical NTM isolates were examined using the availability of the isolates to us at the time. The data generated from the analyses of these 15 pilot samples build provide the justification, along with the large number of environmental samples in this study, for larger, well-controlled clinical and environmental isolate analyses in the future. |
| Quantitative variables | 11 | Explain how quantitative variables were handled in the analyses. If applicable, describe which groupings were chosen and why  Response: N/A |
| Statistical methods | 12 | (*a*) Describe all statistical methods, including those used to control for confounding |
|  |  | (*b*) Describe any methods used to examine subgroups and interactions |
|  |  | (*c*) Explain how missing data were addressed |
|  |  | (*d*) If applicable, describe analytical methods taking account of sampling strategy |
|  |  | (*e*) Describe any sensitivity analyses  Response: The results generated from this study have shown feasibility of such a study, but also identified important limitations. These finding will be used to help design larger and more comprehensive studies linking clinical and environmental aspects of NTM lung disease which we hope to conduct. |
| Results | | |
| Participants | 13* | (a) Report numbers of individuals at each stage of study—eg numbers potentially eligible, examined for eligibility, confirmed eligible, included in the study, completing follow-up, and analysed |
|  |  | (b) Give reasons for non-participation at each stage |
|  |  | (c) Consider use of a flow diagram  Response: We believe these points are not applicable to this study based on the justification we provide above in #10. |
| Descriptive data | 14* | (a) Give characteristics of study participants (eg demographic, clinical, social) and information on exposures and potential confounders |
|  |  | (b) Indicate number of participants with missing data for each variable of interest  Response: Patient metadata was absent from the original submission. During this time of revision, we were able to collect the age and gender of the 15 patients from who clinical isolates were used. This new data is presented in Table 4 and discussed in the text lines 1063-1073 (tracked version). |
| Outcome data | 15* | Report numbers of outcome events or summary measures  Response: N/A |
| Main results | 16 | (*a*) Give unadjusted estimates and, if applicable, confounder-adjusted estimates and their precision (eg, 95% confidence interval). Make clear which confounders were adjusted for and why they were included |
|  |  | (*b*) Report category boundaries when continuous variables were categorized |
|  |  | (*c*) If relevant, consider translating estimates of relative risk into absolute risk for a meaningful time period  Response: a) Confidence intervals were used to report the mean age of the NTM patients for the NTM clinical isolates used in this study; line 1068 tracked version. #16 b and c are N/A. |
| Other analyses | 17 | Report other analyses done—eg analyses of subgroups and interactions, and sensitivity analyses  Response: N/A |
| Discussion | | |
| Key results | 18 | Summarise key results with reference to study objectives  Response: Included in revision; lines 1144-1158, tracked version. |
| Limitations | 19 | Discuss limitations of the study, taking into account sources of potential bias or imprecision. Discuss both direction and magnitude of any potential bias  Response: Limitations are addressed in the revision; lines 1328-1381, tracked version. |
| Interpretation | 20 | Give a cautious overall interpretation of results considering objectives, limitations, multiplicity of analyses, results from similar studies, and other relevant evidence  Response: We have revised the manuscript thoroughly to omit over-statements of findings and provided study limitations. One example in this regard is the use of “pilot samples” to describe the use of the 15 clinical isolates. Additional examples can be found in the revision, lines 1151, 1338-1341, tracked version. |
| Generalisability | 21 | Discuss the generalisability (external validity) of the study results  Response: While we recognize the limitations of this particular study, we discuss the importance and usefulness of these data for future studies, lines 1383-1414, tracked version. |
| Other information | | |
| Funding | 22 | Give the source of funding and the role of the funders for the present study and, if applicable, for the original study on which the present article is based  Response: Although this study itself was unfunded, we include acknowledgements of authors’ funding that supported their roles in the project. |

*Give information separately for exposed and unexposed groups.

Other: Portions of this manuscript have been presented as an oral presentation at the 2016 American Thoracic Society International Conference in San Francisco, California and at the 2016 American Society for Microbiology (ASM) Microbe Conference, Boston, Massachusetts in a poster presentation.

**Note:** An Explanation and Elaboration article discusses each checklist item and gives methodological background and published examples of transparent reporting. The STROBE checklist is best used in conjunction with this article (freely available on the Web sites of PLoS Medicine at http://www.plosmedicine.org/, Annals of Internal Medicine at http://www.annals.org/, and Epidemiology at http://www.epidem.com/). Information on the STROBE Initiative is available at www.strobe-statement.org.
