# Supplementary material for: Environmental Nontuberculous Mycobacteria in the Hawaiian Islands
Source: PLoS Negl Trop Dis. 2016 Oct 25;10(10):e0005068. doi: 10.1371/journal.pntd.0005068 (PMC5079566; doi:10.1371/journal.pntd.0005068)
Supplement: S1 Table — Mycobacteria isolates derived from Hawaiian Island and Continental U.S.A. clinical and household specimens. Species identification and NCBI GenBank accession numbers are provided for each isolate. (PDF) [file pntd.0005068.s002.pdf]

Isolate/Strain	Species	Location	Accession #
12-2-Sw-A-1	*Mycobacterium porcinum*	Hawaii	KU128541
12-36-Sw-A-2	*Mycobacterium porcinum*	Hawaii	KU128542
12-39-Sw-B-1	*Mycobacterium abscessus*	Hawaii	KU128543
12-45-Sw-A-2	*Mycobacterium abscessus*	Hawaii	KU128544
12-55-Sw-B-1	*Mycobacterium paraffinicum*	Hawaii	KU128545
12-56-S-1-2	*Mycobacterium intracellulare*	Hawaii	KU128546
12-57-Sw-A-1	*Mycobacterium abscessus*	Hawaii	KU128547
12-57-Sw-B-1	*Mycobacterium gordonae*	Hawaii	KU128548
12-7-S-1-3	*Mycobacterium chimaera*	Hawaii	KU128549
12-57-S-1-2	*Mycobacterium flavescens*	Hawaii	KU128550
12-58-Sw-B-1	*Mycobacterium abscessus*	Hawaii	KU128551
12-58-S-1-1	*Mycobacterium rhodesiae*	Hawaii	KU128552
12-59-Sw-B-3	*Mycobacterium canariasense*	Hawaii	KU128553
12-61-Sw-B-1	*Mycobacterium porcinum*	Hawaii	KU128554
12-62-Sw-A-2	*Mycobacterium porcinum*	Hawaii	KU128555
KAU-12-2-Sw-A-1	*Mycobacterium porcinum*	Hawaii	KU128556
KAU-12-2-Sw-B-5	*Mycobacterium porcinum*	Hawaii	KU128557
KAU-12-2-Sw-B-3	*Mycobacterium porcinum*	Hawaii	KU128558
KAU-12-2-Sw-B-4	*Mycobacterium porcinum*	Hawaii	KU128559
12-9-Sw-B-1	*Mycobacterium porcinum*	Hawaii	KU128560
KAU-12-5-S-1-2	*Mycobacterium alvei*	Hawaii	KU128561
KAU-12-7-Sw-B-1	*Mycobacterium porcinum*	Hawaii	KU128562
KAU-12-9-Sw-B-1	*Mycobacterium porcinum*	Hawaii	KU128563
KAU-12-9-Sw-B-2	*Mycobacterium porcinum*	Hawaii	KU128564
12-22-S-1-1	*Mycobacterium chimaera*	Hawaii	KU128565
12-26-S-1-2	*Mycobacterium gadium*	Hawaii	KU128566
12-30-Sw-A-1	*Mycobacterium abscessus*	Hawaii	KU128567
12-36-Sw-A-1	*Mycobacterium porcinum*	Hawaii	KU128568
12-39-Sw-A-2	*Mycobacterium phocaicum*	Hawaii	KU128569
12-42-Sw-B-2	*Mycobacterium chelonae*	Hawaii	KU128570
12-49-Sw-B-1	*Mycobacterium chimaera*	Hawaii	KU128571
KAU-12-2-Sw-B-2	*Mycobacterium chimaera*	Hawaii	KU128572
KAU-12-5-Sw-A-1	*Mycobacterium chelonae*	Hawaii	KU128573
12-20-Sw-B-2	*Mycobacterium chelonae*	Hawaii	KU128574
12-30-Sw-A-2	*Mycobacterium phocaicum*	Hawaii	KU128575
12-1-SW-A-1	*Mycobacterium chelonae*	Hawaii	KU128576
12-4-S-1-1	*Mycobacterium barrassiae*	Hawaii	KU128577
12-7-SW-A-2	*Mycobacterium gadium*	Hawaii	KU128578
12-9-SW-A-1	*Mycobacterium chelonae*	Hawaii	KU128579
12-9-SW-A-2	*Mycobacterium chelonae*	Hawaii	KU128580
12-9-SW-A-3	*Mycobacterium chelonae*	Hawaii	KU128581

12-9-SW-B-2	*Mycobacterium abscessus*	Hawaii	KU128582
12-9-SW-B-3	*Mycobacterium abscessus*	Hawaii	KU128583
12-20-SW-B-1	*Mycobacterium chelonae*	Hawaii	KU128584
12-24-SW-B-4	*Mycobacterium abscessus*	Hawaii	KU128585
12-26-SW-B-1	*Mycobacterium chelonae*	Hawaii	KU128586
12-26-SW-B-2	*Mycobacterium chelonae*	Hawaii	KU128587
12-39-SW-A-1	*Mycobacterium abscessus*	Hawaii	KU128588
12-39-SW-B-2	*Mycobacterium abscessus*	Hawaii	KU128589
12-41-SW-B-1	*Mycobacterium chelonae*	Hawaii	KU128590
12-41-SW-B-2	*Mycobacterium chelonae*	Hawaii	KU128591
12-43-SW-B-1	*Mycobacterium chelonae*	Hawaii	KU128592
12-43-SW-B-2	*Mycobacterium chelonae*	Hawaii	KU128593
12-45-SW-A-1	*Mycobacterium abscessus*	Hawaii	KU128594
12-48-SW-B-1	*Mycobacterium abscessus*	Hawaii	KU128595
12-48-SW-B-3	*Mycobacterium abscessus*	Hawaii	KU128596
12-48-SW-B-4	*Mycobacterium abscessus*	Hawaii	KU128597
12-50-SW-A-1	*Mycobacterium abscessus*	Hawaii	KU128598
12-53-SW-A-1	*Mycobacterium chelonae*	Hawaii	KU128599
12-53-SW-A-2	*Mycobacterium chelonae*	Hawaii	KU128600
12-54-SW-A-1	*Mycobacterium chelonae*	Hawaii	KU128601
12-54-SW-B-3	*Mycobacterium chelonae*	Hawaii	KU128602
12-60-SW-A-1	*Mycobacterium chelonae*	Hawaii	KU128603
12-60-SW-B-1	*Mycobacterium chelonae*	Hawaii	KU128604
12-62-SW-A-1	*Mycobacterium chelonae*	Hawaii	KU128605
12-44-SW-A-2	*Mycobacterium chimaera*	Hawaii	KU128606
12-44-SW-A-4	*Mycobacterium chimaera*	Hawaii	KU128607
12-48-SW-A-1	*Mycobacterium chimaera*	Hawaii	KU128608
12-48-SW-B-2	*Mycobacterium chimaera*	Hawaii	KU128609
12-49-SW-A-1	*Mycobacterium chimaera*	Hawaii	KU128610
12-49-SW-B-2	*Mycobacterium chimaera*	Hawaii	KU128611
12-50-SW-B-1	*Mycobacterium chimaera*	Hawaii	KU128612
12-50-SW-B-2	*Mycobacterium chimaera*	Hawaii	KU128613
12-54-SW-B-1	*Mycobacterium chimaera*	Hawaii	KU128614
12-54-SW-B-2	*Mycobacterium chimaera*	Hawaii	KU128615
12-55-SW-B-2	*Mycobacterium chimaera*	Hawaii	KU128616
12-55-SW-B-3	*Mycobacterium chimaera*	Hawaii	KU128617
12-56-SW-A-1	*Mycobacterium chimaera*	Hawaii	KU128618
12-56-SW-B-1	*Mycobacterium chimaera*	Hawaii	KU128619
12-57-SW-B-2	*Mycobacterium chimaera*	Hawaii	KU128620
12-58-SW-A-1	*Mycobacterium chimaera*	Hawaii	KU128621
12-58-SW-B-2	*Mycobacterium chimaera*	Hawaii	KU128622
12-61-SW-A-1	*Mycobacterium chimaera*	Hawaii	KU128623
12-61-SW-A-2	*Mycobacterium chimaera*	Hawaii	KU128624

KAU12-3-SW-A-1	*Mycobacterium chimaera*	Hawaii	KU128625
KAU12-10-SW-A-1	*Mycobacterium chimaera*	Hawaii	KU128626
KAU12-10-SW-A-2	*Mycobacterium chimaera*	Hawaii	KU128627
MOL12-1-SW-B-1	*Mycobacterium chimaera*	Hawaii	KU128628
MOL12-4-SW-A-1	*Mycobacterium chimaera*	Hawaii	KU128629
MOL12-4-SW-A-2	*Mycobacterium chimaera*	Hawaii	KU128630
12-42-SW-B-3	*Mycobacterium abscessus*	Hawaii	KU128631
12-2-SW-A-2	*Mycobacterium porcinum*	Hawaii	KU128632
12-2-S-1-1	*Mycobacterium marseillense*	Hawaii	KU128633
12-7-SW-A-1	*Mycobacterium gordonae*	Hawaii	KU128634
12-16-SW-A-1	*Mycobacterium phocaicum*	Hawaii	KU128635
12-16-SW-A-2	*Mycobacterium phocaicum*	Hawaii	KU128636
12-26-S-1-1	*Mycobacterium interjectum*	Hawaii	KU128637
12-29-S-1-2	*Mycobacterium colombiense*	Hawaii	KU128638
12-44-SW-A-3	*Mycobacterium paraffinicum*	Hawaii	KU128639
12-53-SW-A-3	*Mycobacterium chelonae*	Hawaii	KU128640
12-57-S-1-1	*Mycobacterium arupense*	Hawaii	KU128641
KAU12-1-S-1-1	*Mycobacterium septicum*	Hawaii	KU128642
KAU12-3-SW-A-2	*Mycobacterium porcinum*	Hawaii	KU128643
KAU-12-9-SW-A-1	*Mycobacterium porcinum*	Hawaii	KU128644
BLGD-12-4-SW-B-1	*Mycobacterium longobardum*	Hawaii	KU128645
12-7-S-1-2	*Mycobacterium chimaera*	Hawaii	KU128646
12-56-S-1-1	*Mycobacterium chimaera*	Hawaii	KU128647
12-29-S-1-1	*Mycobacterium colombiense*	Hawaii	KU128648
12-2-SW-B-1	*Mycobacterium chimaera*	Hawaii	KU128649
12-2-SW-B-2	*Mycobacterium chimaera*	Hawaii	KU128650
12-2-SW-B-3	*Mycobacterium chimaera*	Hawaii	KU128651
12-16-SW-B-1	*Mycobacterium chimaera*	Hawaii	KU128652
12-17-SW-A-1	*Mycobacterium chimaera*	Hawaii	KU128653
12-20-SW-A-1	*Mycobacterium chimaera*	Hawaii	KU128654
12-21-SW-A-1	*Mycobacterium gordonae*	Hawaii	KU128655
12-22-SW-A-1	*Mycobacterium chimaera*	Hawaii	KU128656
12-24-SW-A-1	*Mycobacterium chimaera*	Hawaii	KU128657
12-24-SW-B-2	*Mycobacterium chimaera*	Hawaii	KU128658
12-25-SW-B-1	*Mycobacterium chimaera*	Hawaii	KU128659
12-25-SW-B-2	*Mycobacterium chimaera*	Hawaii	KU128660
12-25-S-1-1	*Mycobacterium marseillense*	Hawaii	KU128661
12-25-S-1-2	*Mycobacterium marseillense*	Hawaii	KU128662
12-25-S-1-3	*Mycobacterium marseillense*	Hawaii	KU128663
12-27-SW-A-1	*Mycobacterium chimaera*	Hawaii	KU128664
12-28-SW-A-1	*Mycobacterium chimaera*	Hawaii	KU128665
12-29-SW-A-1	*Mycobacterium chimaera*	Hawaii	KU128666

12-31-SW-B-1	*Mycobacterium chimaera*	Hawaii	KU128667
12-32-SW-B-1	*Mycobacterium chimaera*	Hawaii	KU128668
12-32-SW-B-2	*Mycobacterium chimaera*	Hawaii	KU128669
12-32-SW-B-3	*Mycobacterium chimaera*	Hawaii	KU128670
12-33-SW-A-1	*Mycobacterium chimaera*	Hawaii	KU128671
12-35-SW-A-1	*Mycobacterium chimaera*	Hawaii	KU128672
12-38-SW-A-1	*Mycobacterium chimaera*	Hawaii	KU128673
12-38-SW-A-2	*Mycobacterium chimaera*	Hawaii	KU128674
12-40-SW-A-1	*Mycobacterium chimaera*	Hawaii	KU128675
12-40-SW-A-2	*Mycobacterium chimaera*	Hawaii	KU128676
12-42-SW-A-1	*Mycobacterium chimaera*	Hawaii	KU128677
12-42-SW-A-2	*Mycobacterium chimaera*	Hawaii	KU128678
12-42-SW-A-3	*Mycobacterium chimaera*	Hawaii	KU128679
12-43-SW-A-1	*Mycobacterium chimaera*	Hawaii	KU128680
12-44-SW-A-1	*Mycobacterium chimaera*	Hawaii	KU128681
AH10	*Mycobacterium chimaera*	Hawaii	KU128682
AH11	*Mycobacterium chimaera*	Hawaii	KU128683
AH12	*Mycobacterium chimaera*	Hawaii	KU128684
AH13	*Mycobacterium chimaera*	Hawaii	KU128685
AH14	*Mycobacterium farcinogenes*	Hawaii	KU128686
AH15	*Mycobacterium chimaera*	Hawaii	KU128687
AH16	*Mycobacterium chimaera*	Hawaii	KU128688
AH17	*Mycobacterium marseillense*	Hawaii	KU128689
AH18	*Mycobacterium chimaera*	Hawaii	KU128690
AH02	*Mycobacterium intracellulare*	Hawaii	KU128691
AH20	*Mycobacterium timonense*	Hawaii	KU128692
AH21	*Mycobacterium chimaera*	Hawaii	KU128693
AH22	*Mycobacterium chimaera*	Hawaii	KU128694
AH24	*Mycobacterium intracellulare*	Hawaii	KU128695
AH25	*Mycobacterium chimaera*	Hawaii	KU128696
AH03	*Mycobacterium chimaera*	Hawaii	KU128697
AH04	*Mycobacterium intracellulare*	Hawaii	KU128698
AH05	*Mycobacterium chimaera*	Hawaii	KU128699
AH06	*Mycobacterium chimaera*	Hawaii	KU128700
AH07	*Mycobacterium marseillense*	Hawaii	KU128701
AH08	*Mycobacterium chimaera*	Hawaii	KU128702
AH09	*Mycobacterium intracellulare*	Hawaii	KU128703
KAU-12-5-S-1-1	*Mycobacterium sp. I1*	Hawaii	KU128704
12-6-S-1-1	*Mycobacterium sp. 2*	Hawaii	KU128705
12-6-S-1-3	*Mycobacterium sp. 3*	Hawaii	KU128706
JS010.1	*Mycobacterium chimaera*	Continental USA	KU144826
JS010.2	*Mycobacterium chimaera*	Continental USA	KU144827
JS082.1	*Mycobacterium chimaera*	Continental USA	KU144828

JS082.2	*Mycobacterium chimaera*	Continental USA	KU144829
MA28463.1	*Mycobacterium chimaera*	Continental USA	KU144830
MA28463.2	*Mycobacterium chimaera*	Continental USA	KU144831
MA32872.1	*Mycobacterium chimaera*	Continental USA	KU144832
MA32872.2	*Mycobacterium chimaera*	Continental USA	KU144833
MA4156.1	*Mycobacterium chimaera*	Continental USA	KU144834
MA4156.2	*Mycobacterium chimaera*	Continental USA	KU144835
MA4163.1	*Mycobacterium chimaera*	Continental USA	KU144836
MA4163.2	*Mycobacterium chimaera*	Continental USA	KU144837
MA5205.1	*Mycobacterium chimaera*	Continental USA	KU144838
MA5205.2	*Mycobacterium chimaera*	Continental USA	KU144839
MA5324.1	*Mycobacterium chimaera*	Continental USA	KU144840
MA5324.2	*Mycobacterium chimaera*	Continental USA	KU144841
MA5359	*Mycobacterium chimaera*	Continental USA	KU144842
MA5670	*Mycobacterium chimaera*	Continental USA	KU144843
MA6378	*Mycobacterium chimaera*	Continental USA	KU144844
MA6533	*Mycobacterium chimaera*	Continental USA	KU144845
MA7129	*Mycobacterium chimaera*	Continental USA	KU144846
MA7233	*Mycobacterium chimaera*	Continental USA	KU144847
MA7294.1	*Mycobacterium chimaera*	Continental USA	KU144848
MA7294.2	*Mycobacterium chimaera*	Continental USA	KU144849
MA7320.1	*Mycobacterium chimaera*	Continental USA	KU144850
MA7320.2	*Mycobacterium chimaera*	Continental USA	KU144851
MA7907	*Mycobacterium chimaera*	Continental USA	KU144852
MA7916.1	*Mycobacterium chimaera*	Continental USA	KU144853
MA7916.2	*Mycobacterium chimaera*	Continental USA	KU144854
MA7953.1	*Mycobacterium chimaera*	Continental USA	KU144855
MA7953.2	*Mycobacterium chimaera*	Continental USA	KU144856
P32P1.1	*Mycobacterium chimaera*	Continental USA	KU144857
P32P1.2	*Mycobacterium chimaera*	Continental USA	KU144858

Table S1. Mycobacteria isolates derived from Hawaiian and Continental USA clinical and household specimens. Species identification and NCBI GenBank accession numbers are provided for each isolate.

Isolate/Strain	Species	Location	Accession #
AP025	*Mycobacterium abscessus*	USA	EU090064.1
AP038	*Mycobacterium abscessus*	USA	EU090063.1
ATCC 19977	*Mycobacterium abscessus*	USA	JF346872.1
ATCC 23003	*Mycobacterium abscessus*	USA	AY262741.1
BD	*Mycobacterium abscessus*	Korea	NZ_AHAS00000000.1
CCUG 48898	*Mycobacterium abscessus*	France	NZ_AP014547.1
CIP104536	*Mycobacterium abscessus*	France	EU109292.1
CIP104536T	*Mycobacterium abscessus*	France	AY147164.1
CIP108297	*Mycobacterium abscessus*	France	EU254721.1
CRM 0020	*Mycobacterium abscessus*	Brazil	CP012044.1
CRM 0273	*Mycobacterium abscessus*	Brazil	NZ_ATFQ00000000.1
FI-09338	*Mycobacterium abscessus*	Italy	HM807414.1
FI-09380	*Mycobacterium abscessus*	Italy	HM807434.1
FI-08197	*Mycobacterium abscessus*	Italy	HM807419.1
FI-07134	*Mycobacterium abscessus*	Italy	EU370524.1
GO-06	*Mycobacterium abscessus*	Brazil	FJ859894.1
5S-0304	*Mycobacterium abscessus*	USA	NZ_AKTX00000000.1
IAL014	*Mycobacterium abscessus*	Brazil	HQ404274.1
M24	*Mycobacterium abscessus*	Malaysia	NZ_AJLY00000000.2
P01	*Mycobacterium abscessus*	Brazil	FJ590435.1
PCH-017	*Mycobacterium abscessus*	USA	NC_018150.2
UC22	*Mycobacterium abscessus*	Korea	CP012044.1
6	*Mycobacterium chimaera*	France	EU770576.1
CIP 107892	*Mycobacterium chimaera*	France	GQ153309.1
DSM 446232	*Mycobacterium chimaera*	Germany	EF521908.1
FI-09331	*Mycobacterium chimaera*	Italy	HM807412.1
ATCC 19237	*Mycobacterium chelonae*	USA	EU109289.1
ATCC 35752	*Mycobacterium chelonae*	France	CP010946.1
CIP 104535	*Mycobacterium chelonae*	France	EU109286.1
CIP 104535T	*Mycobacterium chelonae*	France	AY147163.1
D10	*Mycobacterium chelonae*	France	EU109287.1
D8	*Mycobacterium chelonae*	France	EU109290.1
FI-08088	*Mycobacterium chelonae*	Italy	FJ418059.1
FI-09001	*Mycobacterium chelonae*	Italy	HM807422.1
FI-09279	*Mycobacterium chelonae*	Italy	HM807417.1
FI-09307	*Mycobacterium chelonae*	Italy	HM807411.1
PCH-038	*Mycobacterium chelonae*	USA	JN400396.1
QIA-37	*Mycobacterium chelonae*	Korea	KF910163.1
QIA-52	*Mycobacterium chelonae*	Korea	KF910170.1

U4	*Mycobacterium chelonae*	France	EU109291.1
U5	*Mycobacterium chelonae*	France	EU109288.1
08-283	*Mycobacterium porcinum*	USA	EU597591.1
ATCC 49939	*Mycobacterium porcinum*	USA	JN682044.1
ATCC BAA-328	*Mycobacterium porcinum*	USA	JN682045.1
CIP 105392	*Mycobacterium porcinum*	France	AY262737.1
CST-9.1	*Mycobacterium porcinum*	China	JF508476.1
FI-08029	*Mycobacterium porcinum*	Italy	FJ418054.1
MF-114	*Mycobacterium porcinum*	USA	JN682043.1
MF-205	*Mycobacterium porcinum*	USA	JN682046.1
MF-3176	*Mycobacterium porcinum*	USA	JN682049.1
MF-2612	*Mycobacterium porcinum*	USA	JN682048.1

Table S2. Type strain and representative isolate rpoB sequences of *M. abscessus*, *M. chelonae*, *M. chimaera*, and *M. porcinum* from NCBI. Isolation location and NCBI GenBank accession numbers are provided for each sequence.
